# Gender Differences Among Academic Radiation Oncology National Institutes of Health (NIH) Funding Recipients

**DOI:** 10.7759/cureus.28982

**Published:** 2022-09-09

**Authors:** George Mutwiri, Roshini Kulanthaivelu, Joanna Yuen, Mehwish Hussain, Marc Jutras, Curtiland Deville, Reshma Jagsi, Faisal Khosa

**Affiliations:** 1 Radiology, University of Calgary, Calgary, CAN; 2 Radiology, University of British Columbia, Vancouver, CAN; 3 Public Health, Imam Abdurrahman Bin Faisal University, Dammam, SAU; 4 Radiation Oncology and Molecular Radiation Sciences, Johns Hopkins University, Baltimore, USA; 5 Radiation Oncology, University of Michigan, Ann Arbor, USA; 6 Bioethics and Social Sciences in Medicine, University of Michigan, Ann Arbor, USA

**Keywords:** academic medicine, radiation oncology, research productivity, gender equity, nih grants

## Abstract

Purpose

The purpose of our study was to evaluate National Institutes of Health (NIH) funding recipients between 2016 and 2019 to determine if there was an association between gender, research productivity, academic rank, leadership positions, and post-graduate awards.

Materials and Methods

The NIH Research Portfolio Online Reporting Tools Expenditure and Results (RePORTER) website was used to retrieve data for grants in Radiation Oncology from 2016-2019. Demographics and profiles of awardees were retrieved from institutional websites, LinkedIn, and Doximity. Publication metrics were collected through the Scopus database. Mann-Whitney U tests and chi-square analyses were performed to compare and determine associations between gender and other variables.

Results

Three hundred and forty radiation oncology principal investigators (PIs) were included in this study, of whom 76% were men. Of the 776 total NIH grants awarded, 62% of the grants had a sole male PI and 1% had two or more PIs in which the contact PI and co-PI were women. Between the genders of PIs in this sample, there was no significant difference in highest academic rank, leadership positions (i.e., chair, director, founder, president, and other), and post-graduate honors and awards. Total publications, years of active research, h-index, and m-index were higher amongst men in the professor category but were largely similar between genders in the associate and assistant professor categories.

Conclusions

The results demonstrate that most NIH grants in radiation oncology were awarded to men. Strategies that increase women in radiation oncology (RO), as well as those that increase NIH grants amongst women may also increase the prevalence of women in senior academic ranks and leadership positions.

## Introduction

Across academic medicine as a whole, women are less likely to attain full professorship and ﬁll senior leadership positions [1-4]. This disparity is marked in radiation oncology (RO), in which 28% of RO faculty were women yet only 14% of professors and chairpersons were women across 82 RO departments in the United States (US) in 2015 [5]. Potential factors responsible for the disparity in career advancement in academic medicine may include explicit or implicit bias, sexism, sexual harassment, gendered expectations for family care responsibilities, and the exclusion of female role models and mentors [6-10]. In contrast to the more direct explicit bias, implicit bias is composed of unconscious preferences. The Trix and Psenka study showed that in medicine, men were more often viewed as researchers than women [11]. Since RO is considered a highly technical, research-driven specialty, these unconscious biases may be more pronounced in this discipline. One of the most critical factors driving career progression in academic medicine is research productivity, which is often measured using the h-index, as it provides a quantitative assessment of a scientist's research output and signiﬁcance [12]. H-index is deﬁned as the number of papers published by an author (h) that have at least (h) a number of citations [12]. Holliday et al. demonstrated that men in RO had an overall higher h-index than women [5]. However, citation indices were similar when adjusted for academic rank, indicating that differences in research productivity were therefore likely reﬂective of the higher percentage of men in senior academic ranks [5]. Furthermore, there was an under-representation of primary female authors in high-impact medical journals across medical specialties, which may contribute to lower citation rates and thereby impede career advancement [13]. Additionally, Dworkin et al. have demonstrated that there are significant gender imbalances in citationality [14]. Moreover, a review of the literature has shown that gender bias may exist in NIH funding decisions in academic radiology [15].

Gender disparity also prevails amongst the recipients of academic awards, as across various specialties there is a predilection for granting research and recognition awards from medical societies to men [16-18]. This can be significant for career advancement, as award recipients have greater opportunities for networking and enhancing their professional reputation. Knoll et al. have shown gender disparity exists in RO awards, whereby between 2005-2017, only two of the 32 (6.3%) American Society for Radiation Oncology (ASTRO) gold medals were awarded to women [19].

In the current study, we examined the association between gender, research productivity, academic rank, leadership position, highest academic degree, geographic location, and post-graduate honors and awards amongst RO investigators who had received National Institutes of Health (NIH) funding between 2016-2019.

## Materials and methods

This retrospective analysis was exempt from Research Ethics Board review as it comprised non-identifiable, publicly available data. The methodology of this study has been validated in several recent publications [20-27]. Data from all NIH-funded projects categorized as Radiation-Diagnostic/Radiation-Oncology between 2016 and 2019 were collected from the NIH Research Portfolio Online Reporting Tools Expenditure and Results (RePORTER) website (https://projectreporter.nih.gov/reporter.cfm). The number of grants received, name, geographic location, and academic institutions associated with each principal investigator (PI) were obtained. Diagnostic Radiology PIs were manually excluded so that only grants awarded to PIs appointed to RO departments were included in the current analysis. For grants with multiple PIs, the PI for our study was deﬁned as the “corresponding PI”. The co-PI was categorized as female if there was ≥1 female PI listed. Exclusion criteria included graduate students as PIs, medical students as PIs, residents as PIs, research engineers as PIs, PIs solely employed in the private sector, and deceased PIs.

Gender, degree, highest academic rank (i.e., professor, associate professor, assistant professor, other), leadership role, and postgraduate honors and awards for each PI were obtained via academic institutional websites, LinkedIn, and Doximity. Gender was determined by the preferred personal pronoun used in the PI’s institutional proﬁle. Leadership roles included both academic, government, and private sector positions and were categorized into a chair, director, founder, president, and other. Postgraduate honors and awards were deﬁned as any major public accolade acknowledging a PI’s academic achievement. These major awards included awards from professional or research societies, universities, research institutions, and community organizations. Awards were further categorized into teaching, research, clinical, lifetime achievement, and other. Years of active research, the total number of publications, the total number of citations, and the m-index were collected from the Scopus database. If an author had multiple proﬁles, the proﬁle with the highest h-index was used. M-index was also calculated based on this data. Data collection, veriﬁcation, discrepancy resolution, and categorization were performed between April 2019 and May 2021.

SPSS statistical software (IBM Corp. Released 2017. IBM SPSS Statistics for Windows, Version 25.0. Armonk, NY: IBM Corp) was used for statistical analysis. Signiﬁcance levels were set at p < .05. Categorical variables such as gender, education, highest academic position, and teaching awards were presented as frequency and percentages. Median with interquartile range was computed for discrete variables i.e., numbers of academic positions, leadership rank, and awards. Mann-Whitney U test was used to determine signiﬁcance among gender differences in the number of academic positions, publications, leadership roles, post-graduate honors and awards, and citation indices. Bivariate chi-square analyses were used to evaluate correlations between gender, academic rank, leadership, and post-graduate awards. One sample chi-square test was used to check the equal distribution of grants awarded to each gender. These comparisons were stratiﬁed by educational degree and highest academic position of authors. Tableau Desktop software (Tableau Software, LLC, Seattle, WA, version 2020.3) was used to create a map of the geographic distribution of PIs.

## Results

**Figure 1:**
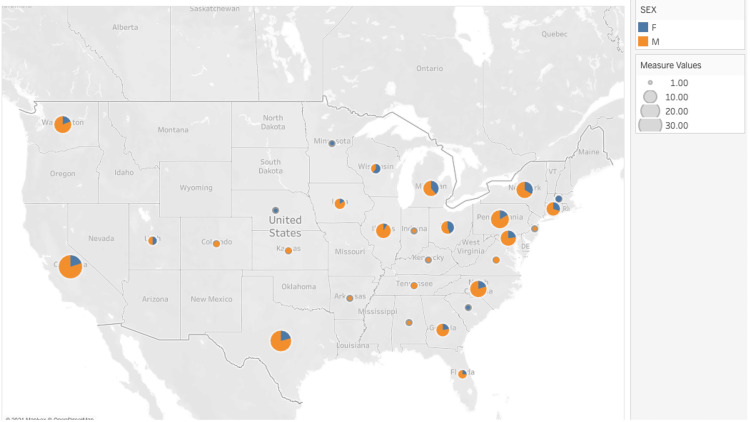
Distribution of radiation oncology principal investigators in the United States. The markers are placed according to the state of each university, and the size represents the total number of principal investigators within each state. The proportion of women and men are displayed in blue and orange, respectively.

There were 340 RO PIs that met our study’s inclusion criteria, 76% of whom were men. Throughout the majority of the US (30 states), more grants were awarded to men than women (Figure 1 ). By contrast, in Nebraska, Minnesota, South Carolina, Wisconsin, and Massachusetts, most grant recipients were women. Most PIs had PhDs (n=247, 72.6%), 63 (18.5%) had MD degrees, 24 (7.1%) had dual doctorates (i.e., MD, Ph.D.) while the remaining six PIs had other degrees (i.e., DVM, DDS, MBBS, PharmD, MB, MA, MSc, etc.). Approximately half of the PIs held the academic rank of Professor (n=180, 52.9%) while nearly one-third were Associate Professor (n=110, 32.4%). Forty-ﬁve PIs (13.2%) were Assistant Professors, and the remaining five held other academic positions (i.e., instructor, fellow, etc.).

Observed differences by gender in academic proﬁles

Table 1 illustrates no significant gender difference in the academic rank of the grant awardees. Female and male PIs had a similar distribution in the academic ranks of professor, associate professor, assistant professor, and others. The median number of academic positions held for both men and women was 1 (IQR=2) and there was no signiﬁcant difference in the number of academic positions between men and women (P=0.852). Bivariate analysis revealed no signiﬁcant differences in academic degrees attained by gender (P=0.238).

**Table 1:** Academic profiles of men and women radiation oncology principal investigators. * : DVM, DDS, MBBS, PharmD, MB, MA, MSc. ** : Instructor, research scientist, fellow.

		Women	Men	P Value
		n	n	
Education	PhD	65 (79.3%)	181 (70.4%)	0.238
	MD, PhD	3 (3.7%)	22 (8.6%)	
	MD	13 (15.9%)	49 (19.1%)	
	Other*	1 (1.2%)	5 (1.9%)	
Highest Academic Position	Professor	43 (52.4%)	137 (53.1%)	0.871
	Associate Professor	26 (31.7%)	84 (32.6%)	
	Assistant Professor	11 (13.4%)	34 (13.2%)	
	Other**	2 (2.4%)	3 (1.2%)	
	Yes	11 (13.4%)	34 (13.2%)	

Observed differences by gender in research productivity

**Figure 2:**
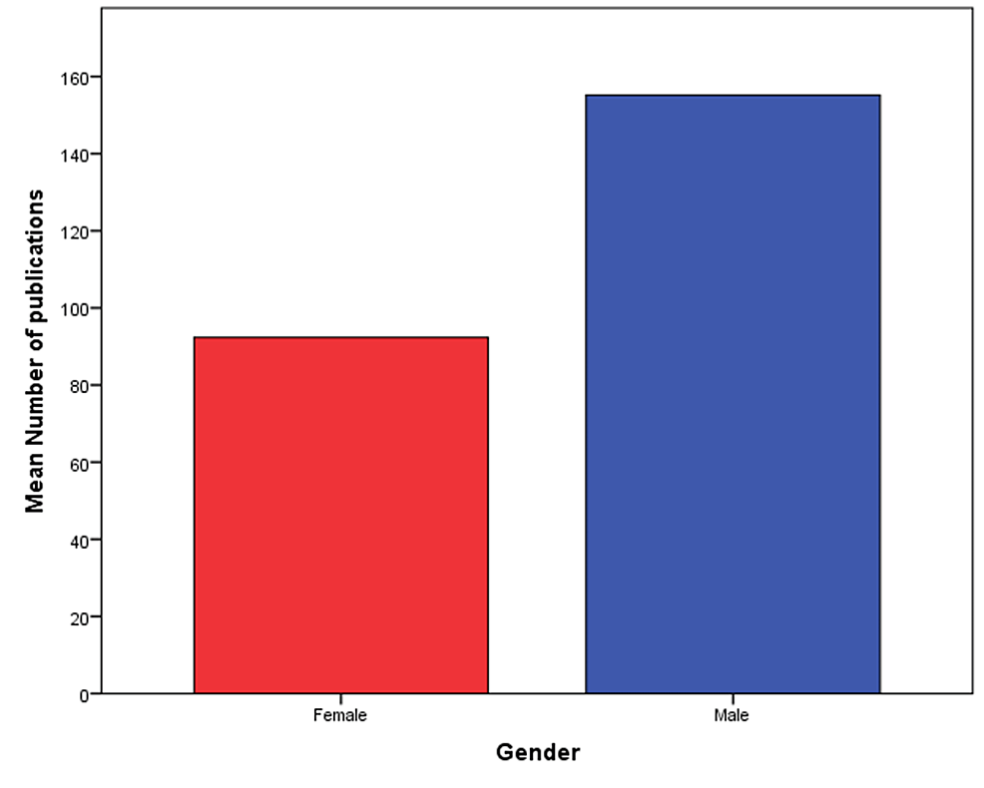
Error bar showing the mean number of publications by men and women radiation oncology prinicipal investigators.

The mean number of publications was 92.3 for women and 155.2 for men (Figure 2). Men and women had almost equal years of active research (Table 2). Men had more citations than women (P=0.032). The mean h-index for men was 37.2, compared to 30.2 for women (P = 0.014). Similarly, women had an m-index 0.2 lower than men (P = 0.014). After stratifying by educational degree, we found a signiﬁcant difference in the average number of publications between men and women with PhDs (P<0.0001), but not amongst MDs and dual doctorates. Men with PhDs authored 143.7±133.9 publications on average while women with PhDs authored on average 83.7±57.0 publications.

**Table 2:** Mean number of publications, years of active research, and citation indices of men and women radiation oncology principal investigators.

	Female	Male
Number of PIs	82	257
Years of active research	22.9±6.4	23.3±6.2
Number of citations	5163.4±6035.8	7700.9±9805.2
H-index	30.2±15.4	37.2±21.8

**Figure 3:**
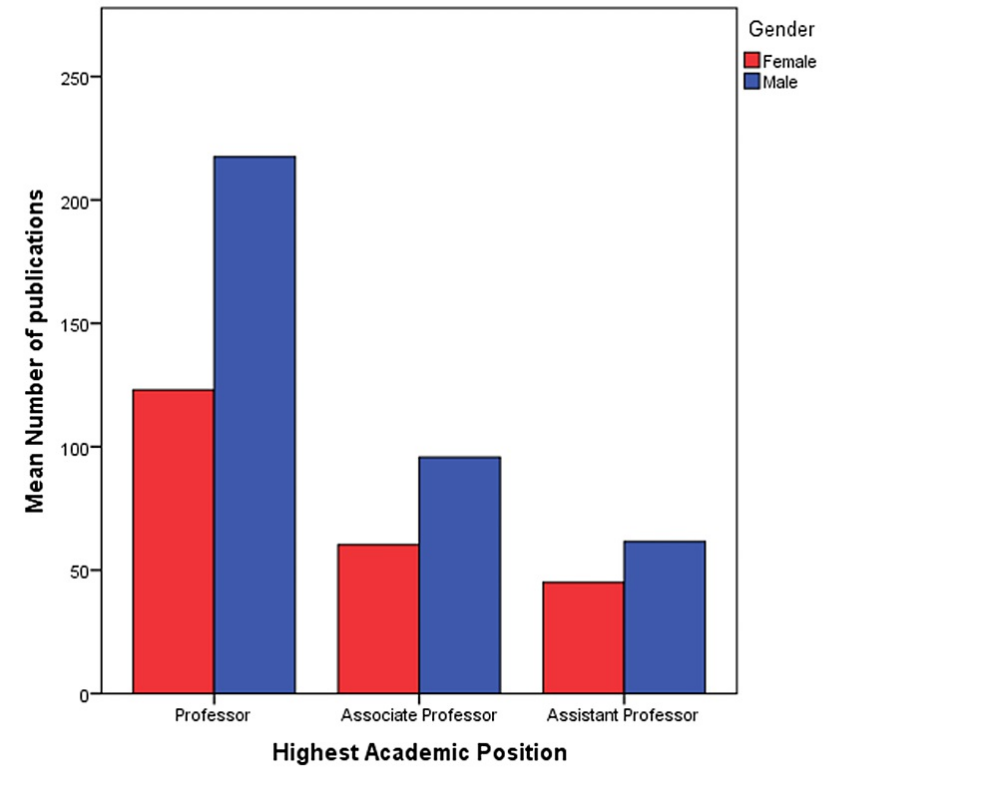
Error bar showing the mean number of publications by men and women radiation oncology principal investigators stratified by highest academic position.

Years of active research, number of citations, h-index, and m-index were not signiﬁcantly different for male and female authors with PhDs, MDs, or dual doctorates. Stratiﬁed analysis based on academic position revealed that amongst professors, the mean number of publications of men was 217.5, compared to 123.1 for women (P<0.0001). In the associate professor category, men had signiﬁcantly more publications than women (Figure 3), however, their citation indices were not signiﬁcantly different. The number of citations, h-index, and m-index was also signiﬁcantly higher for men in this category (Table 3).

**Table 3:** Mean number of publications, years of active research and citation indices of men, and women radiation oncology principal investigators stratified by highest academic position.

		Female	Male	P Value
Professor	Number of publications	123.1±81.0	217.5±159.0	<0.001
	Years of active research	26.8±5.4	27.1±3.7	0.473
	Number of citations	7083.3±6991.3	11686.0±11625.1	0.003
	H-index	37.3±16.6	49.2±22.2	0.001
	M-index	1.4±0.6	1.8±0.7	0.001
Associate Professor	Number of publications	60.3±33.1	95.7±57.8	0.002
	Years of active research	19.2±4.3	20.1±5.6	0.421
	Number of citations	2846.8±2455.4	3682.9±4249.6	0.913
	H-index	23.1±5.6	25.8±11.2	0.331
	M-index	1.3±0.4	1.3±0.5	0.836
Assistant Professor	Number of publications	45.0±20.1	61.6±50.0	0.579
	Years of active research	16.6±3.3	16.4±4.7	0.750
	Number of citations	2052.9±1720.7	2270.1±2572.9	0.958
	H-index	18.8±6.1	19.0±8.3	0.895
	M-index	1.1±0.3	1.2±0.5	0.958

Observed differences by gender in leadership positions and post-graduate honors and awards

The mean number of leadership positions held by PIs was 1.5±2.0, for which the average numbers of chair, director, founder, president, and other positions were 0.5±1.0, 0.7±1.0, 0.03±0.2, 0.05±0.2, and 0.3±0.9, respectively. The number of leadership positions held by women ranged from one to 12, while men ranged from 1-17. The distribution of the number of leadership positions between men and women was not signiﬁcantly different (P=0.556). Each PI received an average of 2.7±4.4 postgraduate awards and honors. The most awards and honors received were in research (2.0±3.8), followed by clinical (0.2±1.5), teaching (0.2±0.7), lifetime achievement 0.07±0.5, and others 0.2±0.6. There was no signiﬁcant difference in the total number of postgraduate awards and honors received among men and women PIs (P=0.725). There was a signiﬁcant difference in clinical awards, with men receiving a maximum of 16 compared to only two by women (0.049). There was no signiﬁcant difference in research, teaching, lifetime achievement, or other awards.

Observed differences by gender in grants

There was a total of 776 grants awarded to RO PIs and the majority of grants had a single male PI (n=481, 62%), followed by a single female PI (147, 19%). Of the remaining 19% (n=148), which had both a contact PI and co-PI named, 71 (9%) had men in both positions, 40 (5%) had a female contact PI with a male co-PI, 28 (4%) had a male contact PI with female co-PI, and nine (1%) had both female contact PI and co-PI. 

## Discussion

This study comprehensively examines how gender relates to academic rank, research productivity, and post-graduate honors and awards, amongst NIH funding awardees in RO departments. Previous studies have shown that women were underrepresented in academic RO departments, particularly in leadership positions, and our study complements and expands upon prior research [28].

Among major radiological societies, studies have shown there remains a significant underrepresentation of women in leadership awards and an overrepresentation of women in teaching awards in comparison to their overall presence in the discipline [29]. This may reflect biases in the award selection and evaluation process that does not mitigate gendered stereotypes and acknowledge longstanding systematic biases and privileges in academic medicine. In our study, we included awards and honors granted by research societies, universities, research institutions, and community organizations in addition to those granted by medical societies. Examples of such honors include Fellow of ASTRO and the Cancer Research Foundation Young Investigator Award. However, our study found no significant difference between genders in the number of postgraduate awards and honors received amongst NIH grant recipients. Our study found that men overall had significantly more publications and citations, a higher h-index, and a higher m-index than women. However, when stratiﬁed by academic rank, differences in citation indices were only present in the academic ranks of professors. This phenomenon does not seem to be present across US RO departments, where publication metrics amongst women and men professors were similar [5]. Perhaps this difference is due to the difference in population, as the majority of PIs who received NIH grants in the present study were PhDs. Interestingly, male associate professors had more publications than women yet with similar h- and m-indices. This suggests that women in this category publish more frequently cited papers to yield comparable citation metrics despite having fewer publications overall. Furthermore, this data indicates that research productivity is similar between genders among early-career RO NIH grant-funded PIs. Nevertheless, given that full professors did show higher citations and h-indices, it is possible that the impact of citationality manifests itself further down along the career trajectory.

This study showed that more grants were awarded to men than women throughout many states within the US, as might be expected given women’s underrepresentation within the discipline of RO overall. Amongst this sample of successful grant applicants, there were no signiﬁcant gender differences in the number of leadership positions, post-graduate awards and honors, and early-career research productivity. As more men receive NIH grants and previous studies have shown that women may require higher amounts of grant funding to achieve similar leadership positions, this may pose a signiﬁcant obstacle in the career progression of women in academic RO [30]. Additionally, in the related discipline of hematologic oncology, women also receive fewer NIH grants when compared to men [31]. This further reinforces the need for gender equity in oncologic research as a whole.

Limitations

Using the current methodology, we were not able to study how explicit and implicit biases might influence the measures assessed. Additionally, gender was determined by the personal pronoun present in each PI’s institutional profile; of note, there were no instances in which a non-binary pronoun was used. However, this may not be entirely representative as some PI's may have non-binary gender identities, which may not be captured within the limitations of their standardized institutional profiles. Moreover, data was collected from publicly available sources which may not be updated and therefore may not reflect the PI's current academic title. In situations where institutional websites were insufficient, webpages such as Doximity and LinkedIn were used to determine the PI’s current position, institution, and honors and awards. Additionally, both private and academic institutions vary in their organizational structure and nomenclature of positions. Categorization was performed based on naming (i.e., chair, director, founder, president, and other), which is not standardized across institutions. Additionally, co-PI was categorized as female if ≥1 female PI was listed for grants with multiple PI's. This may lead to an overestimation of the number of female PIs. There are also inherent limitations to the Scopus database. “Years of active research” is calculated from the first study indexed in Scopus and does not consider career breaks such as parental leave, which may lead to an overestimation of a researcher’s years of continuous research. Furthermore, Scopus does not include articles published prior to 1996 when calculating the h-index. Finally, the RePORTER database only lists RO departments affiliated with a US medical school. Therefore, data from RO departments not associated with medical schools were not included. Also, some departments which conduct RO research (e.g., radiation medicine, human oncology) may not be classified as RO by the RePORTER database, and therefore may not be captured.

## Conclusions

Our study demonstrates that 76% of RO PIs who received NIH grants were men, which may contribute to the paucity of women in the senior-most positions of inﬂuence and authority in RO academic departments. Male full professors had more publications, citations, higher h-indices, and higher m-indices than women in our study sample. However, citation indices were similar between the genders amongst associate professors and assistant professors. There was no signiﬁcant gender difference in the number of post-graduate honors and awards, academic rank, or the number of leadership positions in this sample of PIs. Strategies should be aimed towards attracting and facilitating women into RO training, their retention in academic RO, and provision of resources to ensure their success in academic pursuits including NIH grants.
